# Remodelling of North Staffordshire Infirmary

**Published:** 1913-09-13

**Authors:** 


### REMODELLING OF NORTH STAFFORDSHIRE INFIRMARY.

### The Raising and Results of a Successful Appeal.

Colonel A. H. Heath, who retired from the presidency of the North Staffordshire Infirmary, Hartshill, Stoke-on-Trent, in January 1913, chiefly, we believe, because he felt strongly that the important position of president ought not to be held for several successive years by one individual, was appointed president in the first instance in November 1909. His three years of office have been marked by great changes in the methods of management of the hospital and by much-needed additions and alterations to the buildings of the institution.

At the commencement of 1911 the new rules of the institution, prepared by the careful labours of a strong sub-committee under the chairmanship of Mr. C. E. Bullock, and afterwards revised by the general committee, were submitted to the governors and agreed upon. One of the most important of the changes introduced by these rules was an arrangement whereby eight of the members of the general committee were to be elected by the Hospital Saturday governors, and eight were to be elected by the establishment governors, each of whom had himself been elected by the subscribing workpeople at each of the establishments. The total strength of the general committee is forty-two, consisting of the above-mentioned sixteen members, with fourteen elected by the remaining governors other than establishment and Hospital Saturday governors, and twelve *ex officio* members, including seven senior members of the honorary medical staff.

During the year 1910—while the new rules were being formulated—a special appeal was made to the district to subscribe the large sum of £35,000 for the purpose of carrying out the additions and alterations to the buildings of the institution in accordance with the suggestions made by a special sub-committee appointed to report on these matters. The chairman of this sub-committee was Mr. W. D. Spanton, F.R.C.S., one of the honorary consulting surgeons. Mr. Spanton left the district in August 1911, and was thus unable to see all the suggestions of his sub-committee carried out, but the greater part of the structural additions and alterations mentioned in that sub-committee's report, together with certain other items of building found afterwards to be necessary, have now been completed or are in a fair way to completion. This portion of the work of the hospital has been and is under the direct supervision of the buildings committee, the chairman of which is Mr. J. Taylor Howson.

Thanks largely to the energy and ability of the then president, combined with the assistance rendered by the *Staffordshire Sentinel*, the response to the appeal for the special building fund was immediate. The sum actually promised has amounted to about £34,750, and of this very little remains unpaid at the moment, while if the value of certain additional gifts mentioned later under "Children's Ward" be taken into account, the sum of £35,000 asked for has been more than subscribed. It is also of the greatest interest to note that about £15,000 of this sum has been subscribed absolutely voluntarily by the workpeople at establishments throughout the district by means of 1d. or ½d. per week contributions, in many cases in addition to a similar contribution sent annually to the general maintenance fund.

Every effort has been made by the committee, under the leadership of Colonel Heath, to hurry on the completion of the work, but care has been taken in every instance to get, as far as possible, the utmost value for the money expended. The first improvements to be completed were the erection of a new laundry, fully equipped with modern machinery supplied by Messrs. Manlove, Alliott and Co., of Nottingham; the provision of new boiler and engine houses and new workshops, and the mounting of two additional Cornish boilers (the gift of Colonel Heath); the installing of an entirely new system of heating and hot-water supply, by Messrs. Saunders and Taylor, of Manchester, on the calorifier system; and the provision of additional external fire mains and hydrants. The total cost of these items was about £5,700.

The new out-patient department is now virtually complete and is in use, and comprises a large waiting hall, capable of seating about 280 patients, surrounded by consulting rooms such as one would expect to find in the out-patient department of a large and up-to-date hospital. In addition to medical and surgical consulting rooms there are the following: a room for the ear and throat department, and another for the ophthalmic department; an *x*-ray department fitted with modern appliances for examination and treatment; a theatre, anæsthetising room, and recovery rooms; a dispensary and dispensary stores, with other rooms for various purposes and the necessary offices.

The flooring practically throughout is of terrazzo. The heating is on the "Reck" system. The architects were Messrs. Young and Hall, and the building has been erected under the direct supervision of Mr. Keith D. Young. The total cost of the building, with its furniture and fittings, will approximate to £12,000.

In addition to the out-patient department, the following important additions and alterations have been carried out:—

Renewals of and repairs to block of building known as the Albert Wards. Floor of one ward taken up and relaid in terrano. Alterations to kitchen and sister's room. Entire remodelling of sanitary arrangements; providing large completely tiled bathrooms for each ward, and tiled lavatories with proper cut-off to sanitary tower. Cost about £1,600. Completed and in use.

Practically entire renewing of main kitchen, greatly enlarging and tiling of scullery. Provision of entirely new cooking fixtures (coal-fire range and gas and steam). Completed and in use. Cost about £1,000.

A set of small wards known as the Victoria Wards—until recently used for children—has been entirely remodelled internally to provide the administrative portion, and a ward for temporary isolation.

An entirely new ward has been built on to the old building, the size of which is 96 feet long by 26 feet wide, and is divided into two compartments, one containing ten and the other twenty cots, by a partition consisting entirely of glass in large panes. A broad verandah runs along the south side of the building, covered as to half its width. French windows open on to verandah, so that cots may be wheeled out from the ward. There is a sanitary tower at each end of the ward on the south side, away from the main hospital buildings. Each of the two portions of the ward, and also the isolation ward, could be nursed and administered separately if occasion arose. The whole of the walls of the ward and a dado in the front hall have been tiled at the expense of a generous donor. The tiles are of a soft greenish tint, with egg-shell gloss finish. Panels containing baskets of flowers, hand painted, extend round the whole ward, and a dado and frieze of brighter flowers set off the whole. Practically completed, but not yet in use. The heating is on the "Reck" system.

In addition to bearing the cost of the expensive tiling above described, Mrs. Spibey, of Alsager, provided additional sums for certain other useful additions, and has also provided the thirty cots for the ward, and certain other articles of furniture. Cost, exclusive of gifts above mentioned, about £3,200.

A new wing to the nurses' home, providing an additional twenty-four bedrooms, a large general recreation room, and bathrooms, box rooms, store rooms, etc., is almost completed at a cost of £2,900; while a further £300 has been expended in altering and improving the heating and hot-water apparatus in the older portion of the nurses' home. All the bathrooms throughout the institution are to be remodelled, and two of these have been completed, the walls being tiled to the ceilings and new fixtures being put in. Many of the sanitary annexes were remodelled before 1912, and the remainder will be.

In addition to the above several other important improvements are to be proceeded with immediately; these include additional internal fire hydrants, fire staircases to two wards, alterations to and fitting up of secretarial offices, alterations to resident medical officers' quarters, provision of new ranges in ward kitchens, external painting and pointing.

A few weeks ago a non-resident pathologist was appointed at a salary of £200 per annum, and every effort will be made to hurry along the alterations necessary for the fitting up of a new pathological department. It is also proposed to build an additional operation theatre for in-patients at an early date. The architect for the whole of the structural work at present proceeding, and all that which has been completed during the past four years—with the exception of the new out-patient department—is Mr. Alex. Scrivener, of Messrs. R. Scrivener and Sons, of Hanley. The total cost of all the structural additions and alterations completed since the beginning of 1910, at present being proceeded with, or in immediate contemplation, will be approximately £30,500 to £31,000.

In addition to providing this large sum of money for the special building fund the people of North Staffordshire have generously responded to the call of the committee for increased annual income. For though it is true that the relative position as between the yearly income and the yearly expenditure has not improved, this is due to the fact that more patients have been dealt with, and that greater efficiency has been produced throughout.

The increase in the income of the hospital is almost entirely due to the increase in the contributions from the employees at the collieries, potteries, and other establishments throughout the district whose contributions last year (October 1911 to October 1912) amounted to nearly £7,000 out of a total ordinary income of £13,059.

| Year ending Oct. | 1904 | 1908 | 1909 | 1910 | 1911 | 1912 |
|---|---|---|---|---|---|---|
| Ordinary Income ... | 12,598 | 12,519 | 11,122 | 13,488 | 14,037 | 13,059 |
| Extraordinary Income... | — | 500 | 265 | 381 | 808 | 2,666 |
| Total Income ... ... | 12,598 | 13,019 | 11,387 | 13,869 | 14,845 | 15,725 |
| Ordinary Expenditure... | 11,964 | 12,924 | 13,671 | 13,376 | 15,274 | 16,097 |
| Extraordinary Expenditure ... ... ... | 276 | 475 | 1,519 | 964 | 51 | — |
| Total Expenditure ... | 12,240 | 13,399 | 15,190 | 14,340 | 15,325 | 16,097 |
| No. of In-patients treated to a conclusion ... | 1,917 | 2,239 | 2,183 | 1,946 | 2,262 | 2,370 |
| No. of Out-patients ... | 13,920 | 14,845 | 16,829 | 15,149 | 16,969 | 18,020 |

The fall in the ordinary income during 1912 was due to the coal strike, which lasted two months.

It will thus be seen that the North Staffordshire Infirmary has very greatly benefited from the great arousing of public interest which took place in the year 1909, coincident with and following the visit to the hospital of Sir Henry Burdett and the articles which appeared in The Hospital, and too much praise cannot be given to Colonel Heath who took the helm at a critical time, and, with the aid of a strong and representative committee, has guided the North Staffordshire Infirmary to the calmer waters of its present position.

In Mr. H. J. Johnson the infirmary has been fortunate in obtaining a president in every way fitted to carry on the work ably begun by Colonel Heath. Mr. Johnson is one of the best known men of business in North Staffordshire, whose knowledge and ability has been always at the service of the Infirmary. It is hoped that Mr. F. J. Harrison, the senior vice-president, who would have taken the presidency this year in the normal course but for a sudden illness, will act as president in the near future.

Mr. Harrison was chairman of the sub-committee whose special duty it was to erect the new children's ward. Just before leaving for his voyage he showed his consideration for the sick of the district by presenting to the infirmary £3,000—£500 towards the equipment of a pathological department and £500 per annum for five years to be used for its maintenance. Though much has been done, much remains to be done; but on the whole the prospects of the North Staffordshire Infirmary seem to be good, if energetic work on the part of the general committee representing all classes of the community can command success.

*** This account should be contrasted with the article which appears on page 704 on "Representation and the Voluntary System." That article discusses the theory and this is a record of practice.

